# Combination AURKA and WEE1 Inhibition Exhibits Efficacy in EGFR or Pan-ERBB Inhibitor–Resistant Head and Neck Squamous Cell Carcinoma

**DOI:** 10.1158/2767-9764.CRC-26-0368

**Published:** 2026-08-13

**Authors:** Flaviane N. Silva, Jong Woo Lee, Theodore T. Nguyen, Fiona M. Goeckel, Tetyana Bagnyukova, Hossein Borghaei, Erica A. Golemis, Barbara A. Burtness

**Affiliations:** 1 Cancer Signaling and Microenvironment Program, Fox Chase Cancer Center, Philadelphia, Pennsylvania.; 2 Drexel University College of Medicine, Philadelphia, Pennsylvania.; 3 Section of Medical Oncology, Department of Internal Medicine, Yale Cancer Center, Yale University School of Medicine, New Haven, Connecticut.; 4 Chestnut Hill College, Philadelphia, Pennsylvania.; 5 Department of Cancer and Cellular Biology, Lewis Katz School of Medicine, Philadelphia, Pennsylvania.

## Abstract

More than 600,000 cases of head and neck squamous cell carcinoma (HNSCC) are diagnosed globally each year. Many HNSCCs overexpress the ERBB family member epidermal growth factor receptor (EGFR), and EGFR inhibitors (EGFRi) and pan-ERBB inhibitors (ERBBi) are clinically active in the treatment of locally advanced, metastatic, or recurrent HNSCC. However, resistance to these inhibitors typically develops, often associated with epithelial–mesenchymal transition (EMT). Aurora kinase A (AURKA), a mitotic regulator with expanded signaling functions in tumors, has been reported to reverse EGFR resistance in EGFR-mutated lung cancer. To identify strategies to overcome resistance in HNSCC, we developed HNSCC cell models that were treatment-naïve parental or selected for resistance to the EGFRi erlotinib or the ERBBi afatinib. The resistant HNSCC models had typically undergone partial EMT, consistent with clinical resistance, associated with upregulation of the AURKA partner protein NEDD9. Synergy of the AURKA inhibitor VIC-1911 with erlotinib or afatinib in parental models was reduced in resistant models in short-term growth assays. In longer-term clonogenic assays and *in vivo*, cells selected for EGFRi/ERBBi resistance showed heightened sensitivity to VIC-1911, contributing to reduced synergy. Based on increased AURKA dependence, we compared the combination of VIC-1911 with adavosertib, an inhibitor of the cell-cycle checkpoint regulator WEE1, in parental and resistant models. These showed a combination effect both *in vitro* and *in vivo* that was retained in ERBBi-resistant xenografts, suggesting the potential value of combined AURKA and WEE1 inhibitor use in patients with ERBBi-resistant HNSCC.

**Significance:**

Treatment resistance is a major source of mortality for HNSCC. This study investigates strategies to overcome resistance to inhibitors of the ERBB family, which are commonly used for the treatment of advanced HNSCC. The results suggest the value of the use of combined AURKA and WEE inhibition in the resistance setting.

## Introduction

There are projected to be 72,680 new cases of head and neck squamous cell carcinomas (HNSCC) in the United States in 2025, associated with ∼16,700 deaths (1). These tumors most commonly occur in the oral cavity, oropharynx, and larynx. Human papillomavirus (HPV) infection and integration of HPV into the genome are significant causes of HPV-associated (HPV^+^) HNSCCs arising from the oropharynx (2), in which carcinogenesis occurs due to p53 and Rb degradation caused by the virally encoded E6 and E7 oncogenic proteins (3, 4). HPV-negative (HPV^−^) tumors more commonly develop from the oral cavity and larynx as a result of chronic exposure to carcinogens, such as alcohol and tobacco, and have a high burden of mutation (5). Although the 5-year survival rate for HNSCC has been increasing, diagnosis with advanced disease remains common and is associated with worse outcomes; in 2023, the 5-year survival rate for localized HNSCC was 86.6%, and it was lower for locally advanced (69.1%) or recurrent/metastatic (39.3%) HNSCC (7).

Standard therapy for recurrent/metastatic HPV^−^ HNSCC includes chemotherapy, targeted therapy, and immunotherapy (6, 7). Targeted inhibitors of cancer signaling proteins have been extensively investigated in HNSCC, as in other cancers. The epidermal growth factor receptor (EGFR), a member of the family of ERBB receptor tyrosine kinases (RTK), activates multiple signaling cascades that promote tumor growth and survival. EGFR is often overexpressed in HNSCC and was one of the first targetable proteins identified as relevant to this disease (8). The EGFR-targeting antibody cetuximab has been, for over two decades, the only validated targeted therapy in HNSCC; is commonly used in HNSCC treatment; and improves response and survival when combined with chemotherapy or radiation (9, 10). However, cetuximab-treated patients often develop resistance through the activation of alternative RTKs (10) and other mechanisms that compensate for EGFR inhibition (11), with many cases of resistance arising from unknown causes. Identifying strategies to overcome resistance to EGFR inhibitors (EGFRi) would yield considerable clinical benefit.

Some approaches in recent trials have included dual antibody and kinase inhibitor EGFR inhibition (12), simultaneously targeting multiple members of the ERBB RTK family (NCT02979977; ref. 13). These have had promising objective response rates, in some cases in patients resistant to initial treatment with cetuximab. Both the EGFR-specific inhibitor erlotinib and the pan-ERBB inhibitor (ERBBi) afatinib have been assessed and have single-agent activity. Both erlotinib and afatinib seem to improve ORR when combined with cetuximab (14). However, neither erlotinib nor afatinib ultimately advanced survival in randomized trials when given in combination with radiation (erlotinib; ref. 15), alone for recurrent/metastatic disease (afatinib; ref. 16), or as maintenance after chemoradiation for high-risk locally advanced HNSCC (afatinib; ref. 17). A major limitation of these studies is that all were biomarker-unselected. However, subsequent analysis of the Machiels trial demonstrated differences in afatinib benefit by HPV status and PTEN expression (18). Overall, given the potential for HER2 or HER3 to rescue cells after EGFR inhibition, the pan-ERBBi afatinib has greater promise, and afatinib has been reported to have activity comparable with that of cetuximab (19). Further insights into mechanisms of pan-ERRB inhibitor resistance remain relevant to potential future treatments for HNSCC, both in refining biomarker patient selection strategies and in designing combination treatments that can dampen activation of adaptive signaling that contributes to cell survival and treatment resistance.

A number of studies have suggested that inhibiting Aurora kinase A (AURKA) is beneficial as a combination treatment with EGFR, including in the setting of EGFR resistance (20–22). In untransformed cells, AURKA functions are primarily restricted to mitosis and reflect activities promoting the normal function of the centrosomes and the mitotic spindle in the cell cycle. These activities are mediated in part through AURKA phosphorylation of its effector PLK1, which in turn increases CDC25 phosphatase dephosphorylation of Y15 on CDK1, allowing the CDK1–CCNB1 complex to initiate mitotic entry. These activities are opposed by the WEE1 kinase, which phosphorylates Y15 and serves as a G_2_–M checkpoint. AURKA is commonly expressed at much higher levels and is more catalytically active in many tumors compared with normal tissues, overcoming the restraint of CDK1/CCNB1 by WEE1. Cells entering mitosis with hyperactive AURKA often develop mitotic abnormalities such as multipolar spindles that lead to genomic instability. Notably, AURKA overexpression in cancer broadens the intracellular distribution of AURKA to include the nucleus and cytoplasm, significantly expanding the range of proteins phosphorylated by AURKA. Studies in various types of cancer have suggested that overexpressed AURKA directly or indirectly promotes the phosphorylation and activity of multiple proteins normally activated by EGFR and its proximal effectors, including AKT, ERK1/2, and others (23). Intriguingly, one report studying lung cancer has identified an adaptive survival program in which cells treated with EGFRis upregulate the AURKA interacting protein TPX2, leading to increased AURKA activity and contributing to resistance to drugs targeting these proteins or EGFR (21).

Both the cell-cycle and non–cell-cycle activities of AURKA in cancers have motivated the exploration of AURKA inhibitors (AURKAi) as potentially useful therapeutic agents. These inhibitors reduce the number of cells capable of entering mitosis; those that do typically have monopolar spindles and other abnormalities that lead to mitotic catastrophe. Although a number of AURKAis have now been assessed in the clinic, these have typically been reported to have low or modest single-agent activity against various forms of cancer (24, 25), and no AURKA-targeting agent has been approved for single-agent, standard-of-care use, with phase III trials failing to show consistent survival benefit (26). These disappointing results have led to a focus on AURKA combination therapies. Exploring AURKA combinations in preclinical studies in HNSCC, we have previously combined the AURKAi alisertib with the WEE1 inhibitor (WEE1i) adavosertib, finding promising activity *in vitro* and in xenografts (27). Combinations of AURKAis and EGFRis are showing promise in studies of EGFR-mutated lung cancer (21) but have not been investigated in HNSCC. Furthermore, none of these combinations have been investigated in the setting of EGFRi-resistant or pan-ERBBi–resistant tumors.

In this study, we have investigated the combination of the AURKAi VIC-1911 with the EGFRi erlotinib, the pan-ERBBi afatinib, or the WEE1i adavosertib, comparing efficacy in sensitive parental models with efficacy in derivative HNSCC models that are erlotinib- or afatinib-resistant. These studies indicate that although AURKA inhibition significantly reduces the growth of EGFRi-resistant or pan-ERBB–resistant models, and AURKA inhibition is synergistic with EGFR or pan-ERBB inhibition in the treatment-naïve setting, synergy is not observed in the resistant setting, in part because of greater inhibition associated with the single-agent use of the AURKAi. In contrast, the AURKAi plus WEE1i combination retains some activity in pan-ERBB–resistant models, suggesting the potential value of this combination in an expanded population of patients.

## Materials and Methods

### Cell culture and drugs

FaDu (CVCL_1218) and Cal27 (CVCL_1107) cell lines were obtained from American Type Culture Collection, and their identities were verified through short tandem repeat profiling performed by IDEXX BioAnalytics. FaDu cells were cultured in EMEM (Corning Life Science, item 10-009-CV, Corning, NY). Cal27 cells were cultured in DMEM (Corning Life Sciences, 50-013-PB), supplemented with 1× GlutaMAX (Thermo Fisher Scientific, item 35050061). All media were supplemented with 10% FBS and 0.1 mg/mL penicillin–streptomycin. *Mycoplasma* contamination testing was routinely performed on both cell lines using the Lonza MycoAlert Mycoplasma Detection Kit (Lonza, LT07-318). For drug treatments, cells were treated with vehicle (0.01% DMSO), VIC-1911 (VITRAC), adavosertib (MedChemExpress, HY-10993), erlotinib (MedChemExpress, E-4997), or afatinib (MedChemExpress, HY-10261).

### Generation of erlotinib- and afatinib-resistant models

FaDu and CAL27 human HNSCC cells with acquired resistance were established by treatment with increasing concentrations of afatinib or erlotinib starting at 10 nmol/L, followed by a stepwise dose escalation of 3 days on/1 day off, up to a maximum of 8 μmol/L for afatinib-resistant cells and 18 μmol/L for erlotinib-resistant cells. Once resistance was established, multiple cell vials were frozen for each resistant cell line. For use in experiments, cells were thawed and allowed to recover in the absence of drug for a week. Cell stocks were then typically split twice a week and maintained under selective drug conditions for up to eight passages and cultured in the absence of drug for 24 hours before experiments; they were then discarded, and a new vial was thawed.

### Cell viability and cell death

Viability assays were performed in technical triplicates in 96-well plates, with cells treated with vehicle (DMSO) or serial dilutions of the indicated drugs for 72 hours. At the end of the drug treatment, 10 μL/well of CellTiter-Blue (Promega, G8081) was added to the media for 2 hours of incubation at 37°C, and then absorbance was determined at 574 nm using a Perkin Elmer EnVision 2102 plate reader. The dose–response curves were built with GraphPad Prism (RRID:SCR_002798), using the nonlinear regression curve fit model called “(log)inhibitor vs. normalized response–variable slope.” The relative IC_50_ (midpoint between top and bottom response) was also calculated based on this model. For synergy analyses, the Loewe additivity model was used in the SynergyFinder 3.0 platform (28). Deviations between observed and expected responses with positive and negative values denote synergy and antagonism, respectively.

For cell death assays, NUCLEAR-ID Blue/Red cell viability reagent (Enzo Life Sciences, ENZ-53005) diluted with 1× PBS (1:1,000 dilution) was used. One hundred microliters per well was added to the wells after 72 hours of incubation with drugs. Cells were incubated for 30 minutes at 37°C, and images were acquired using ImageXpress Micro Confocal (IXM-C, Molecular Devices), and the Multi-Wavelength Scoring module (MetaXpress, Molecular Devices) was used for scoring blue and red (dead) cells. Levels of apoptotic cells were assessed using a FITC Annexin V Apoptosis Detection Kit with propidium iodide (PI; BioLegend, cat. #640914).

### Clonogenic and spheroid growth

For clonogenic growth, cells were seeded into 24-well plates and treated with the indicated drugs for a total of 11 days, with fresh media and drugs added every 3 days. To assess colony survival, cells were fixed with 10% methanol and 10% acetic acid in dH_2_O, followed by staining with 0.2% crystal violet. The total area was calculated using ImageJ (RRID:SCR_003070). For spheroid analysis, cells were seeded in technical triplicates in U-bottom, 96-well plates with a cell-repellent surface (Greiner Bio-One, 650970). After 24 hours, drugs or vehicle were added; after 120 hours of incubation, cell viability was assessed using a CellTiter-Glo 2.0 Cell Viability Assay (Promega, item G9241). Plates were equilibrated at room temperature for 20 minutes. Sixty microliters of CellTiter-Glo per well was added to the plates, followed by 5 minutes of shaking and 25 minutes of incubation at room temperature. After incubation, the luminescent signal was determined using a PerkinElmer EnVision 2102 plate reader.

### Cell-cycle analysis

Cells were treated with the indicated drugs or vehicle for 24 hours, then trypsinized, collected, and fixed in 70% ethanol overnight. After fixation, cells were washed with PBS and incubated with PI/RNase staining buffer (BD Biosciences, 550825) for 15 minutes on ice. Cells were sorted by flow cytometry using a FACSymphony A5 from BD BioSciences, and data were analyzed using FlowJo (RRID:SCR_008520).

### Western blot analysis

Adherent cells were lysed with CellLytic M (Sigma, item C2978) and 1× Halt Protease Inhibitor Cocktail (Thermo Fisher Scientific, item 78429), scraped, and centrifuged at 13,300 × *g* for 30 minutes at 4°C. The Pierce BCA Protein Assay Kit (Thermo Fisher Scientific, 23223) was used to assess the protein concentration of supernatants. Twenty-five micrograms of protein lysates were prepared with Pierce Lane Marker Reducing Sample Buffer 5× (Thermo Fisher Scientific, item 39000) and boiled for 5 minutes at 100°C. Samples were loaded into SDS polyacrylamide gel electrophoresis (SDS-PAGE) Bolt Bis-Tris 4% to 12% (Invitrogen, NW04120BOX). Proteins were transferred overnight at 4°C to a nitrocellulose membrane, which was then blocked with SuperBlock (Thermo Fisher Scientific, 37515). Primary antibodies (Supplementary Table S1) were prepared in SuperBlock at a 1:1,000 dilution. For secondary antibodies, both horseradish peroxidase (HRP; 1:10,000) and alkaline phosphatase (AP; 1:5,000) systems were utilized. When using HRP-conjugated secondary antibody, blots were developed by Immobilon Western Chemiluminescent HRP Substrate (MilliporeSigma, WBKLS0500). The Immun-Star AP Conjugate Substrate Kit (Bio-Rad, item 1705018) was used for AP-conjugated antibodies. In order to detect proteins of different molecular weights, membranes were commonly cut into separate sections and incubated with different primary antibodies. As a result, some panels in this study share the same loading control because they were derived from the same gel. In addition, certain membrane sections were stripped and reprobed sequentially to detect the phosphorylated and total forms. To produce graphs of Western blot data, protein signals were quantified using ImageJ (RRID:SCR_003070) software, and signal intensity was normalized to a loading control. All graphs of Western blot data shown in the study were generated based on at least three independent biological repeats, each independently normalized to loading controls.

### Immunoprecipitation

Cells were lysed as previously described. Twenty-five microliters of protein A/G plus agarose beads (sc-2003, Santa Cruz Biotechnology) was incubated overnight with the primary antibody anti-AURKA (Rabbit, 1:100, Cell Signaling Technology, #3092) or negative rabbit IgG control (Rabbit, 1:100, Cell Signaling Technology, #2729). On the next day, the beads were washed with CellLytic M (Sigma, item C2978) and 1× Halt Protease Inhibitor Cocktail (Thermo Fisher Scientific, item 78429) and resuspended in 500 μg of protein lysates. After overnight incubation at 4°C, the samples were centrifuged at 4,500 *g* for 5 minutes. The supernatant was collected (input control), and the lysate beads were washed twice. The samples were then prepared for Western blotting.

### RNA sequencing and DepMap analysis

About 3 × 10^6^ adherent cells were harvested from 10 cm^2^ plates. After spinning the cells down, the media was completely removed, and the dry pellet was snap-frozen with liquid nitrogen. Cells were stored at −80°C until shipment. Two biological replicates were prepared for each cell model. Samples were shipped to GENEWIZ (RRID:SCR_003177, Azenta Life Sciences), where RNA was extracted from frozen cell pellets. RNA integrity was assessed using TapeStation. RNA with poly(A) selection was used for library preparation and was further subjected to Illumina HiSeq sequencing, generating paired-end reads with a read length of 150 nt. Following sequencing, data quality was assessed by FastQC analysis, and raw sequence data were aligned to the human reference genome GRCh38 using the STAR aligner. Differential gene expression analysis was performed using DESeq2, and functional annotations were obtained from Gene Ontology and gene set enrichment analysis (GSEA). For the differential expression analysis, *P* values and log_2_ fold changes were calculated using the Wald test. A *P* value < 0.05 and log_2_ fold change >1 defined differentially expressed genes. The RNA sequencing (RNA-seq) data generated in this study have been deposited in the NCBI Gene Expression Omnibus (GEO) under the accession number GSE324492.

To identify the profile of pathogenic mutations in the Cal27 and FaDu cell lines, we retrieved data for total and pathogenic mutations from DepMap (https://depmap.org/portal; ref. 29) using the DepMap Public 25Q3 Dataset.

### Xenograft analysis

All mice were housed in a specific pathogen-free environment at the Yale University Animal Facility and treated in strict accordance with a protocol (#2025-20269) approved by the Yale Institutional Animal Care and Use Committee. Six- to eight-week-old female athymic nude (*NU/J*; 002019; RRID:IMSR_JAX:002019) mice purchased from The Jackson Laboratory were used in the cell-derived xenografts (CDX) studies. All mice were allowed access to sterile food and water *ad libitum*. Animal sample size was based on estimations by G* power (RRID:SCR_013726), a two-sided *t* test, and balanced group sizes, and the study was powered to detect large group differences with an effect size in the range of d = 2.4 to 1.5 at 80% power.

For the FaDu parental and afatinib-resistant CDXs, one million cells were subcutaneously injected into both flanks of a nude mouse to establish xenografts. Mice bearing tumors with a mean size of 150 to 300 mm^3^ were randomly grouped and received one of the following treatments: (i) vehicle, (ii) VIC-1911 (30 or 60 mg/kg, daily for 6 days, orally), (iii) adavosertib (120 mg/kg, daily for 6 days, orally), or (iv) combination adavosertib (120 mg/kg) + VIC-1911 (30 mg/kg, daily for 6 days, orally) for 3 weeks. Agents were freshly prepared before dosing: Adavosertib powder was dissolved in 5% DMSO, 40% polyethylene glycol-300 (PHR3336; Sigma-Aldrich), 5% Tween 80 (655207; Sigma-Aldrich), and 50% PBS, and VIC-1911 powder was dissolved in filter-sterilized 0.5% (hydroxypropyl) methyl cellulose (09963; Sigma-Aldrich) in PBS. Monitoring of tumor growth and body weight was conducted blindly. Tumor volume was calculated as follows: (*l* × *w*^2^)/2, where *l* and *w* refer to the largest and smallest perpendicular dimensions at each measurement, respectively, and tumor-bearing mice were monitored daily for 6 days per week. Mice were euthanized when tumor volume was greater than 1,000 mm^3^, or tumor ulceration was observed based on humane euthanasia criteria; for mice that had not reached this endpoint, all were euthanized after 12 days of drug treatment for the VIC-1911 60 mg/kg study and after 21 days of drug treatment for the VIC-1911 plus adavosertib study. Tumor regression, determined by GraphPad Prism (RRID:SCR_002798)–applied multiple linear regression analysis; median tumor volume; and treatment tolerability, were also considered.

### IHC

IHC was performed as previously described (21). Collected tumor tissues were fixed in 10% formalin in PBS overnight and transferred to 70% ethanol. Tissues were further processed with paraffin embedding and sectioning by Yale Pathology Tissue Service (YPTS). Sections were deparaffinized, rehydrated, and subjected to high-temperature antigen retrieval with 10 mmol/L sodium citrate buffer (pH 6.0). Sections were blocked with BLOXALL endogenous blocking solution (SP-6000; Vector Laboratories) and normal goat serum and incubated with primary antibodies against Ki-67 (MIB-1; M7240; RRID:AB_2142367; 1:500; Dako), cleaved caspase 3 (#9664; RRID:AB_2070042; 1:500; Cell Signaling Technology), total AURKA (#14475; RRID:AB_2665504; 1:125; Cell Signaling Technology), or TPX2 (#AB32795; RRID:AB_778561; 1:250; Abcam) overnight. Sections were washed and treated with SignalStain Boost IHC detection reagent (HRP; rabbit; #8114; RRID:AB_10544930 or Mouse; #8125; RRID:AB_10547893; Cell Signaling Technology). HRP-conjugated secondary antibodies were visualized using the SignalStain DAB substrate kit solution (#8059; Cell Signaling Technology) and followed by counterstaining with hematoxylin. The stained tissues were randomly captured using an Revolution microscope (Echo, a Bico company) with 10× automatic positioning. For the scoring of tumor specimens, 7 to 9 pictures per tumor section were acquired and blindly scored according to a scale from 0 to 3.

### Statistical analysis

Data were analyzed using GraphPad Prism (RRID:SCR_002798). Bar graphs display the mean and standard error of the mean (SEM). Comparisons between two groups were calculated using a two-tailed unpaired Student *t* test. For comparisons among three or more groups for *in vitro* experiments, we used one-way ANOVA followed by Tukey multiple comparison *post hoc* test. An adjusted *P* value < 0.05 was considered statistically significant. To test whether the drug treatments *in vivo* affected tumor volume and mouse body weight, we used two-way ANOVA followed by Tukey multiple comparison *post hoc* test. An adjusted *P* value < 0.05 was considered statistically significant.

## Results

### Development of HNSCC cell models with induced resistance to afatinib and erlotinib

**Figure 1.**
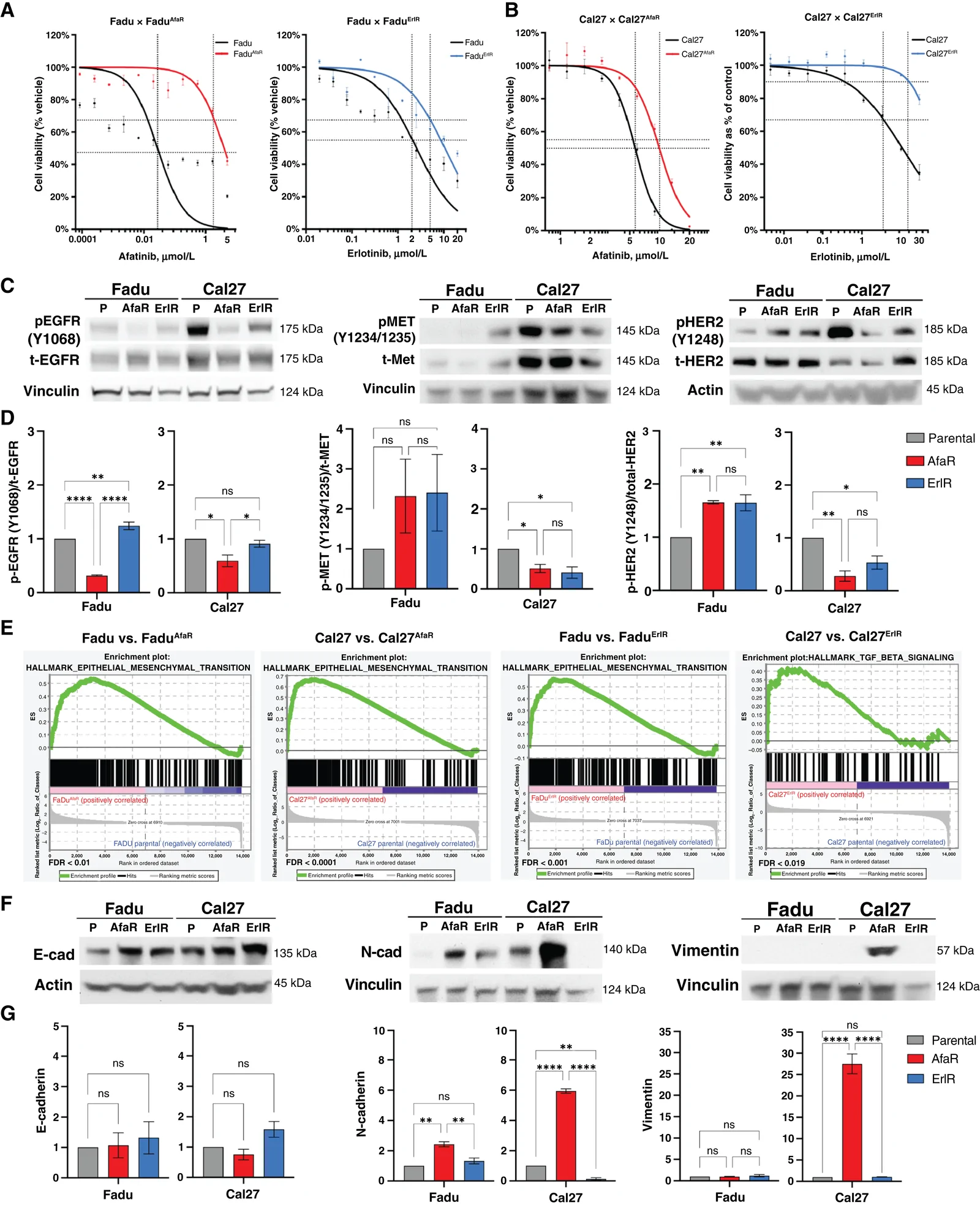
Characterization of ErlR and AfaR HNSCC cell models. **A** and **B,** Dose response curves for (**A**) Fadu and (**B**) Cal27 parental, ErlR, or AfaR cell lines treated with the indicated dose range of afatinib or erlotinib for 72 hours. **C,** Representative Western blot images for active RTKs EGFR, MET, and HER2. **D,** Quantification of Western blots shown in **C**, with values normalized to those in parental cell lines. Graphs display baseline activation relative to total levels of each protein, based on *n* = 3. Note that for some proteins analyzed (e.g., MET), there was a significant intensity difference between FaDu and Cal27 models. For these cases, multiple exposures were taken for the relevant data, and quantification was performed based on primary Western blot data within a linear signal range. **E,** GSEA comparing Fadu and Cal27 AfaR or ErlR vs. Fadu and Cal27 parental cells, as indicated. RNA-seq analysis shown is averaged from two biological replicates. **F** and **G,** Representative Western blot images (**F**) and quantification (**G**) for indicated biomarkers of epithelial identity or EMT, based on *n* = 3 biological repeats. Bar graphs display data normalized for each parental cell line. *P* values are based on one-way ANOVA followed by Tukey multiple comparison test. *, *P* ≤ 0.05; **, *P* ≤ 0.001; ****, *P* ≤ 0.0001. Data are shown as mean ± SEM of three biological replicates. ES, enrichment score.

To compare drug sensitivity profiles in EGFRi or ERBBi-sensitive versus -resistant cells, we developed four new cell models. For this purpose, the FaDu and Cal27 parental cell lines were cultured in gradually increasing doses of erlotinib or afatinib (Fig. 1A and B). Based on CellTiter-Blue and 72-hour drug treatment, afatinib-resistant FaDu (Fadu^AfaR^) cells had relative IC_50_ values of 1.77 μmol/L for afatinib compared with 28 nmol/L for parental FaDu; erlotinib-resistant FaDu (Fadu^ErlR^) cells had relative IC_50_ values of 5 μmol/L for erlotinib compared with 2 μmol/L for FaDu cells. Cal27^AfaR^ had relative IC_50_ values of 9.3 μmol/L for afatinib compared with 5.7 μmol/L for Cal27 parental cells; Cal27^ErlR^ IC_50_ values were 15 μmol/L for erlotinib compared with 3.5 μmol/L for parental Cal27 cells.

The intrinsic level of resistance to afatinib and erlotinib was significantly higher in parental Cal27 than in FaDu cells (3.5 μmol/L versus 28 nmol/L for afatinib and 3.5 μmol/L versus 2 μmol/L for erlotinib). Examination of the mutational profile of these two models based on data in DepMap identified a significant mutational burden in both models, with 73 predicted pathogenic single-nucleotide variants (SNV) in FaDu cells and 61 in Cal27 cells. Although few of these mutations had clear relevance to resistance phenotypes, two SNVs in the *NRAS* gene, resulting in two amino acid changes (R68T and D92N) in the CAL27 model, are of potential interest, as one study has suggested that a D92N mutation studied in conjunction with a G48R mutation in *HRAS* is mildly activating (30), whereas the presence of the R68T mutation has been reported in various cell lines in addition to CAL27, suggesting it may have an activating role. In addition, the baseline expression of cMET was significantly higher in the CAL27 cell model, providing a second potential contributor to higher baseline resistance (Fig. 1C).

Activation of cMET seemed to increase in the FaDu resistant models, although this change was not statistically significant (Fig. 1C and D). On the other hand, AKT activation was unaffected or reduced in three of the four models (with FaDu^ErlR^ being an outlier; Supplementary Fig. S1A and S1B), whereas HER2/ERBB2 activation was modestly elevated in the resistant FaDu models (Fig. 1C and D), and ERK activation trended toward increased activation in resistant CAL27 models (Supplementary Fig. S1A and S1B). As a hypothesis generator for sources of resistance, we analyzed mRNA-seq data for the parental versus resistant cell models (Supplementary Table S2). No pattern of consistent change was identified in the mRNA expression of ERBB genes or their canonical effectors (Supplementary Fig. S1C). GSEA demonstrated that the most characteristic features of three of the four resistant cell models (Fadu^ErlR^, FaDu^AfaR^, Cal27^AfaR^) were a signature for upregulation of genes associated with epithelial–mesenchymal transition (EMT; Fig. 1E; ref. 31), compatible with the known role of EMT in driving clinical therapeutic resistance (32, 33). Western analysis confirmed the upregulation of the mesenchymal marker N-cadherin in three of the four models (but not Cal27^ErlR^), and vimentin was additionally elevated in Cal27^AfaR^ (Fig. 1F and G). However, in spite of this increase in the EMT marker N-cadherin, levels of the epithelial marker E-cadherin did not decrease in these cells; a phenotype compatible with a partial EMT (pEMT) conversion (34, 35). GSEA of the fourth resistant model, Cal27^ErlR^, indicated the greatest elevation of signatures for TGFβ and MYC, also commonly associated with loss of epithelial characteristics, EMT, and drug resistance (Fig. 1E; Supplementary Fig. S1D; refs. 36, 37). Notably, the Cal27^AfaR^ model also displayed a statistically significant elevation of TGFβ signaling, suggesting dominance of this resistance pathway in the Cal27 background (Supplementary Fig. S1E).

### AURKA expression and response to AURKAis in parental, AfaR, and ErlR cells

**Figure 2.**
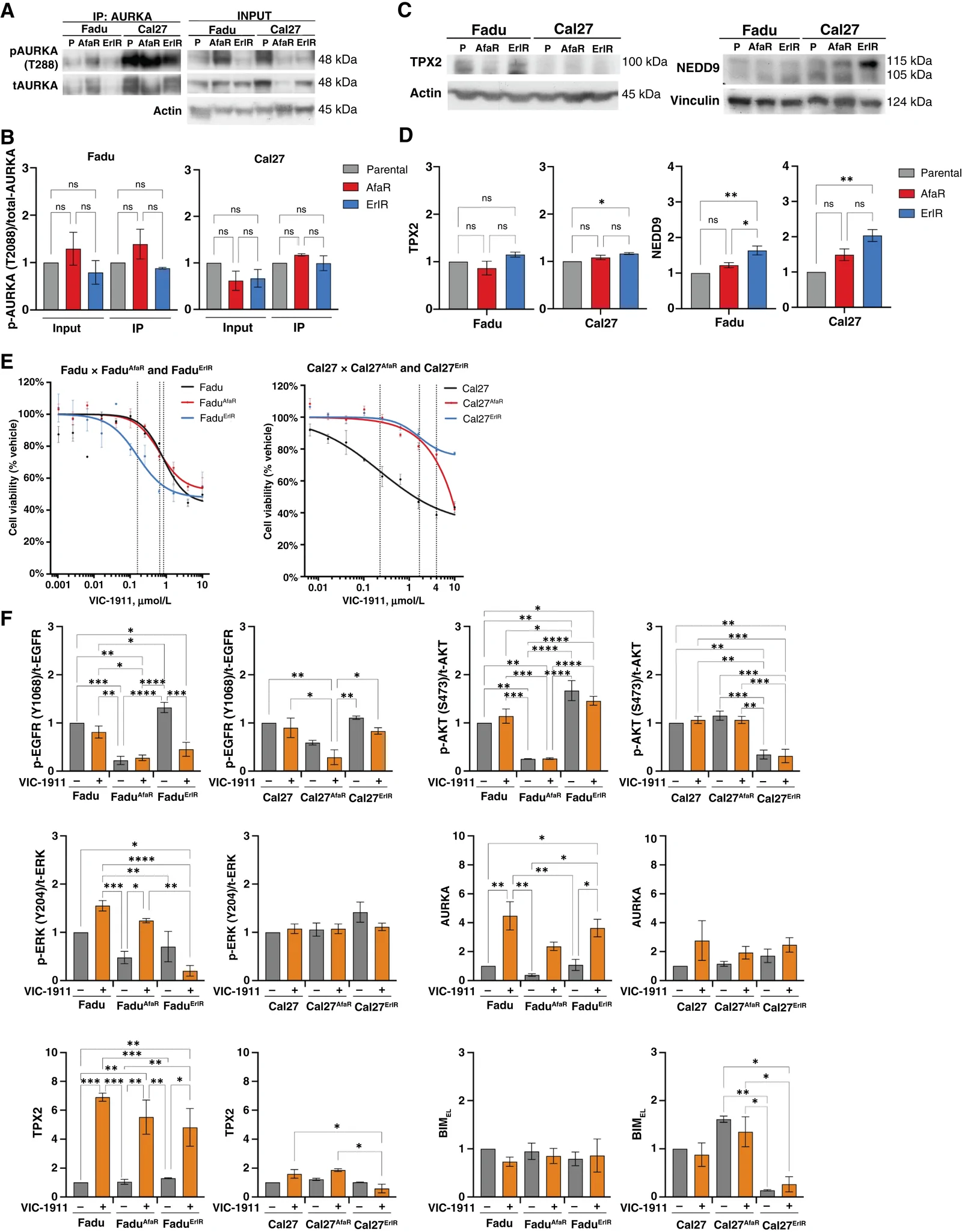
Response of parental, AfaR, and ErlR models to AURKAi monotherapy. **A,** Representative Western blot images showing baseline activation and total levels of AURKA. **B,** Quantification of immunoprecipitation assay probing for phospho-AURKA (T288) and total AURKA protein levels. **C,** Representative Western blot images of TPX2 and NEDD9. **D,** Western blot quantification for baseline levels of TPX2 and NEDD9, with antibodies to total protein. **E,** Dose–response curves for parental, ErlR, and AfaR cell models treated with the indicated dose range of VIC-1911 for 72 hours. **F,** Fadu/Cal27 parental and derived resistant models were treated with vehicle and VIC-1911 (0.6 μmol/L) for 24 hours. Protein level quantification for phospho-EGFR (y1068) was relative to total EGFR, phospho-AKT (S473) relative to total AKT, phospho-ERK (Y204) relative to total ERK, total AURKA, TPX2, and Bim_EL_ relative to the loading control. Bar graphs display data normalized to each parental cell line. All graphs were quantified from at least three independent biological repeats. *P* values are based on one-way ANOVA followed by Tukey multiple comparison test. *, *P* ≤ 0.05; **, *P* ≤ 0.01; ***, *P* ≤ 0.001; ****, *P* ≤ 0.0001. Data are shown as mean ± SEM of three biological replicates.

In EGFR-mutated lung cancer, upregulation of AURKA activity, driven by increased expression of its partner and activator TPX2 (38), has been associated with resistance to EGFRis (21). However, in three of the four AfaR and ErlR models, AURKA expression and activity were not significantly elevated compared with the parental lines (Fig. 2A and B). In further contrast to reports in lung cancer, the expression of TPX2 was not significantly changed in the resistant models (Fig. 2C and D). Elevated expression and hyperphosphorylation of NEDD9, an alternative AURKA partner, have been shown to protect AURKA activity from small molecule inhibition (39) and have a strong association with EMT (40, 41). Interestingly, levels of total or hyperphosphorylated (slower migrating) NEDD9 were increased in the ErlR resistance models; hyperphosphorylation of NEDD9 enhances its capacity to interact with partner proteins, promoting signaling activity and activation of NEDD9 partner proteins (Fig. 2C and D; ref. 42).

To determine whether EGFRi-resistant cell models had altered responses to AURKA inhibition, we compared the IC_50_ for the AURKAi (AURKAi) VIC-1911 in ErlR and AfaR compared with parental models, following 72 hours of drug treatment. These data did not reveal a consistent pattern of AURKAi response based on EGFRi-resistance phenotype, with a relative IC_50_ for VIC-1911 of 0.84 μmol/L in FaDu, 0.66 μmol/L in FaDu^AfaR^, and 0.16 μmol/L in FaDu^ErlR^. In contrast, the relative IC_50_ for the AURKAi was 0.23 μmol/L in Cal27 parental cells, 4 μmol/L in Cal27^AfaR^, and 1.67 μmol/L in Cal27^ErlR^ (Fig. 2E). Notably, analysis of the cell cycle indicated similar or greater accumulation of cells at G_2_–M after 24 hours of VIC-1911 in both resistant and parental cell models, suggesting comparable drug uptake and on-target activity across the parental and derivative models (Supplementary Fig. S2A).

Using Western blot analysis, we examined the signaling response to VIC-1911 treatment in each of the models after 24 hours of treatment (Fig. 2F). In all models, VIC-1911 treatment resulted in elevated levels of total AURKA and strongly induced TPX2; evidence that drug uptake and inhibitory activity for AURKA were comparable in all models. However, VIC-1911 did not consistently affect the levels of active (phosphorylated) EGFR, AKT, or ERK in any cell model, nor did it affect the expression of BIM_EL_, a regulator of apoptosis described as a target of AURKA in lung cancer (21).

### Combined inhibition of EGFR and AURKA in parental and EGFRi-resistant cell models

**Figure 3.**
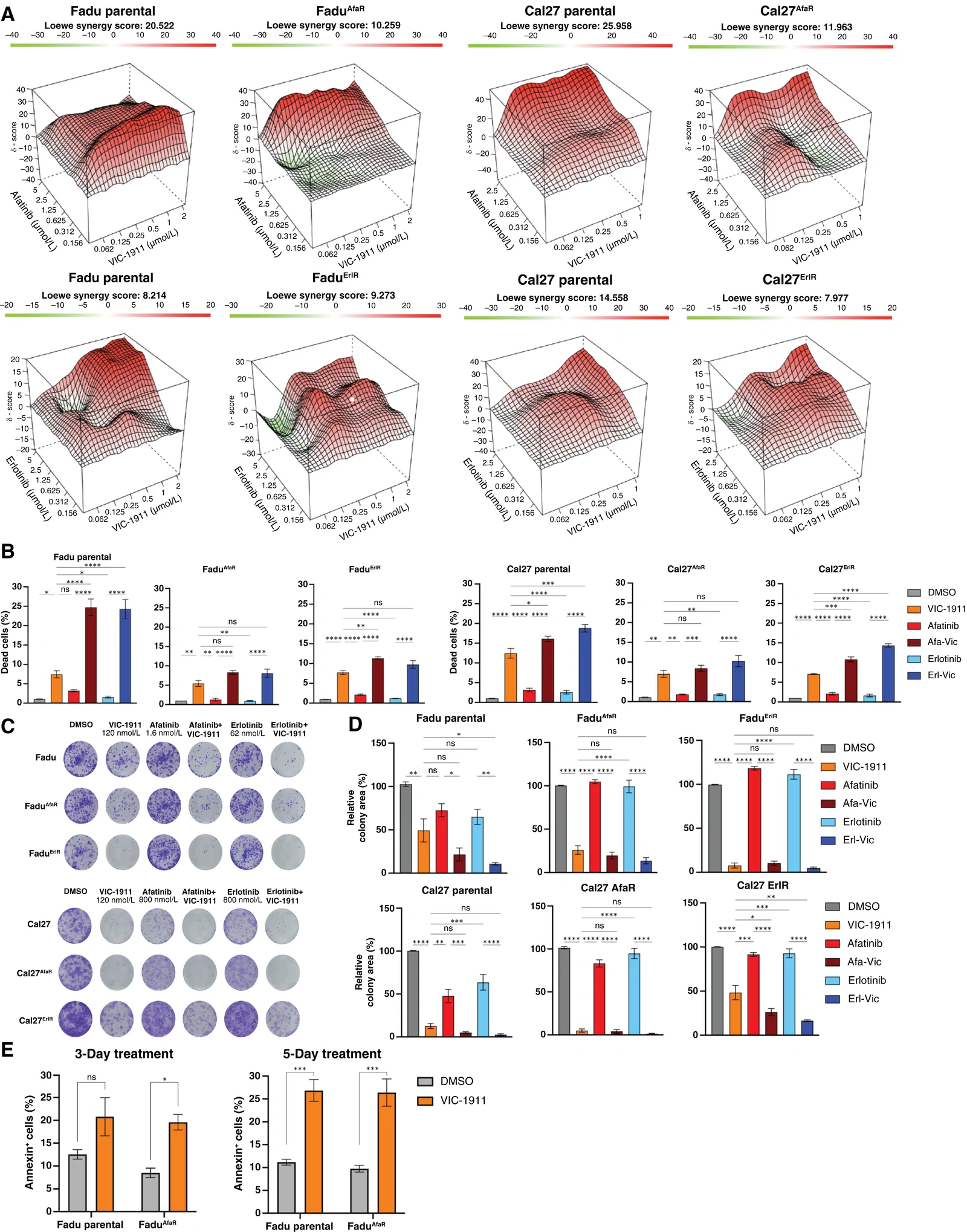
Response of parental, AfaR, and ErlR models to AURKAi combinations with erlotinib or afatinib. **A,** Loewe synergy plot showing synergistic interaction between VIC-1911 and afatinib or erlotinib in FaDu and Cal27 parental, ErlR, and AfaR cell lines treated for 72 hours, *n* = 3. **B,** Quantification of dead cells from the models indicated treated for 72 hours with drugs indicated. FaDu and derived models were treated with vehicle, VIC-1911 (0.6 μmol/L), afatinib (0.2 μmol/L), erlotinib (1 μmol/L), or combination. Cal27 and derived models were treated with vehicle, VIC-1911 (0.6 μmol/L), afatinib (0.8 μmol/L), erlotinib (2 μmol/L), or combination for 24 hours. **C** and **D,** Clonogenic survival assay primary data (**C**) and corresponding (**D**) quantification. Cells were treated with the drug concentration indicated in **C** for 11 days. **E,** Quantification of annexin-positive cells for Fadu parental and Fadu AfaR treated for 3 days (left) and 5 days (right) with VIC-1911 (600 nmol/L). *P* values are based on one-way ANOVA followed by the Tukey multiple comparison test. *, *P* ≤ 0.05; **, *P* ≤ 0.01; ***, *P* ≤ 0.001; ****, *P* ≤ 0.0001. Data are shown as mean ± SEM of three biological replicates.

Evaluation of the combined effect of afatinib with VIC-1911 in the parental cell lines using Loewe analysis of CellTiter-Blue data showed a robust synergistic effect in reducing viability after 72 hours of drug treatment in both FaDu and Cal27 cell models but lesser synergy in the resistant setting (Fig. 3A). Analysis of the effect of cell-cycle compartmentalization indicated comparable outcomes in parental, AfaR, and ErlR models (Supplementary Fig. S2B). Levels of cell death 72 hours after treatment indicated effective killing by the VIC-1911 combination with erlotinib or afatinib in the two parental models that significantly exceeded that induced by each drug alone but a reduced combination effect in the resistant models (Fig. 3B). However, spheroid analysis in low-adhesion plates also indicated limited value of drug combinations versus single-agent use (Supplementary Fig. S2C and S2D).

In contrast to these short-term assays, in a longer term, 12-day clonogenic assay, the combination of VIC-1911 with either afatinib or erlotinib was also more effective than the use of either drug alone in the FaDu parental cell line (Fig. 3C and D). Using the same dose level for each drug for the parental and the two resistant models, none of the EGFRi-resistant models had reduced clonogenic capacity induced by erlotinib or afatinib. In contrast, three out of four of the EGFRi-resistant models displayed heightened sensitivity to AURKA inhibition compared with the parental line, with no additive effect of the drug combination. The greater sensitivity to AURKA inhibition in clonogenic assays likely reflects the accumulation of cell-cycle defects over time in the context of failure of mitotic checkpoints, as reflected in an increasingly abnormal cell-cycle compartmentalization and a higher degree of cell death by 5 days after treatment with VIC-1911 (Fig. 3E; Supplementary Fig. S3).

Analysis of signaling by core EGFR pathway effectors and AURKA-related proteins was conducted 24 hours after drug treatment *in vitro* in the FaDu (Supplementary Fig. S4) and Cal27 (Supplementary Fig. S5) models. Basal levels of EGFR^ph1068^ were depressed in the AfaR-resistant models for both FaDu and Cal27, and treatment with afatinib or erlotinib eliminated discernible activation, but combination with VIC-1911 had no further effect (Supplementary Figs. S4A and S5A). VIC-1911 did not significantly affect levels of AKT^ph473^ or ERK^phT202/Y204^, or total levels of these proteins, in combination with afatinib or erlotinib, in any model (Supplementary Figs. S4B, S4C, S5B, and S5C). Combination with afatinib or erlotinib did not influence VIC-1911 induction of TPX2, or total levels of AURKA or BIM_EL_ (Supplementary Figs. S4D–S4F and S5D–S5F).

### Dual inhibition of AURKA and WEE1 in EGFRi- and ERBBi-resistant HNSCC

As an alternative approach to targeting EGFRi-resistant or pan-ERBBi–resistant cells, we had previously shown that by intensifying the mitotic catastrophe associated with AURKA inhibition, the combined application of AURKAi and WEE1i led to significantly more control of HNSCC cell growth *in vitro* and tumor growth in xenograft models than either agent used alone (27). We investigated whether this combination was effective in the setting of erlotinib- or afatinib-resistant HNSCC, using VIC-1911 in combination with the WEE1i adavosertib.

**Figure 4.**
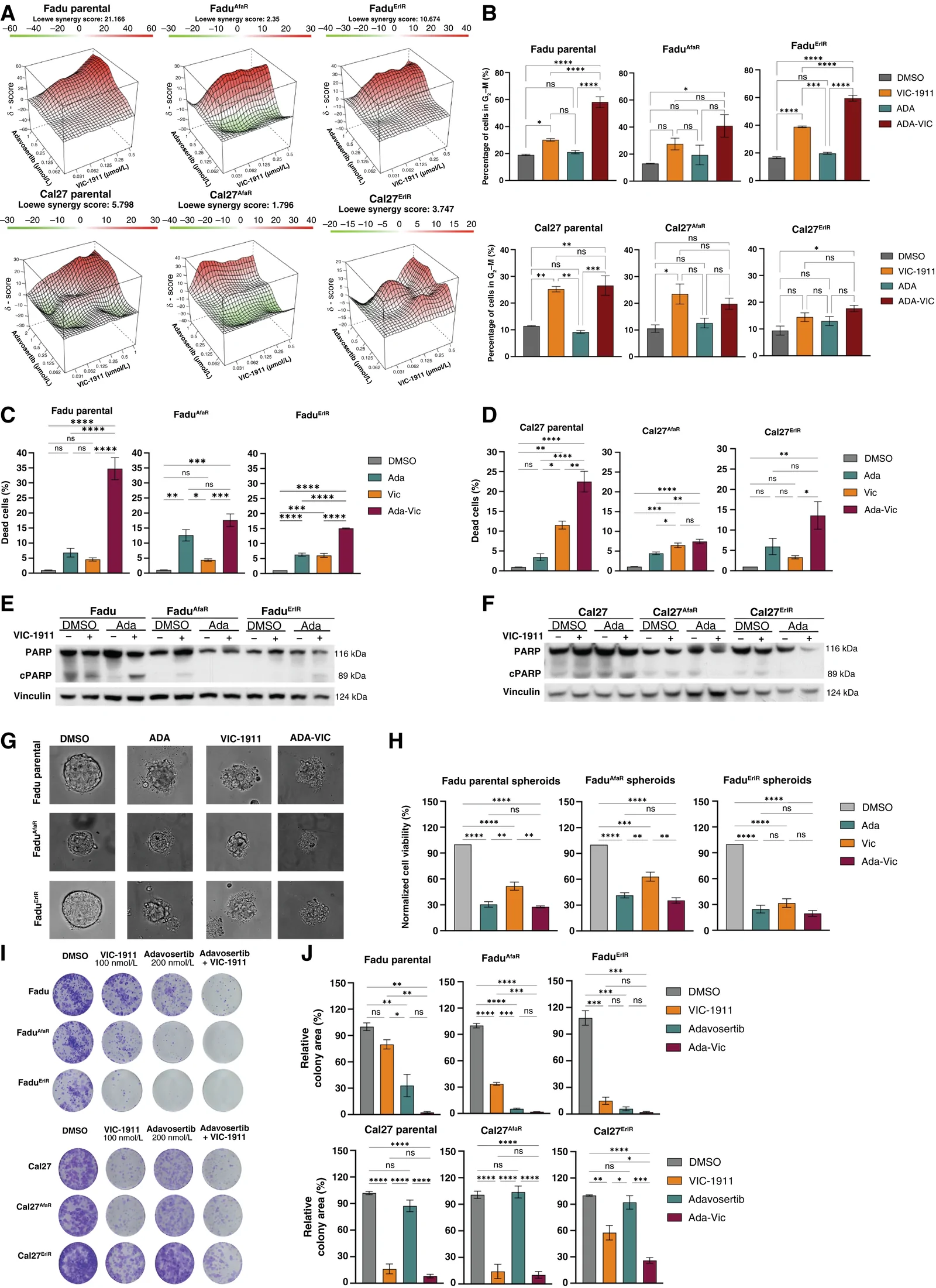
Efficacy of a VIC-1911 combination with adavosertib in parental, AfaR, and ErlR HNSCC cell models. **A,** Loewe synergy plot showing synergistic interaction between VIC-1911 and adavosertib in parental, ErlR, and AfaR cell models treated for 72 hours, *n* = 3. **B,** Percentage of cells in G_2_–M (4N DNA content) for parental, ErlR, and AfaR cell models treated with DMSO, VIC-1911 (0.25 μmol/L), adavosertib (0.5 μmol/L), or combination for 24 hours. **C** and **D,** Quantification of dead FaDu (**C**) and Cal27 (**D**) parental, ErlR, and AfaR cell models treated with vehicle, adavosertib (0.5 μmol/L), VIC-1911 (0.25 μmol/L), or combination for 72 hours (*n* = 3). **E** and **F,** Representative Western blot images showing PARP and cleaved PARP (cPARP) for parental, ErlR, and AfaR cell models treated with DMSO, VIC-1911 (0.25 μmol/L), adavosertib (0.5 μmol/L), or combination for 24 hours, *n* = 3. **G** and **H,** Representative images (**G**) and quantitation of viability based on CellTiter-Glo (**H**) for spheroids of indicated cell models. **I** and **J,** Representative images (**I**) and quantification (**J**) of clonogenic assays for cells treated with the drug concentration indicated in **I** for 11 days. Bar graphs display data normalized to DMSO values of each cell line. *P* values are based on one-way ANOVA followed by the Tukey multiple comparison test. ns, not significant; *, *P* ≤ 0.05; **, *P* ≤ 0.01; ***, *P* ≤ 0.001; ****, *P* ≤ 0.0001. Data are shown as mean ± SEM of three biological replicates.

Loewe synergy analysis in the FaDu and Cal27 series of parental, AfaR, and ErlR models using CellTiter-Blue to assess viability following 72 hours of drug treatment indicated synergy in the FaDu parental model, an additive effect in the Cal27 models, and a significant reduction of single-agent and combination effects in the resistant models (Fig. 4A). Cell-cycle compartmentalization analysis performed using the same concentration of drugs in all models indicated a strong interaction between VIC-1911 and adavosertib in causing G_2_–M arrest and accumulation of >4N cells, particularly in the FaDu models (Fig. 4B; Supplementary Fig. S6A). Measurements of cell death at the same time point showed a highly significant increase in dead cells following treatment with the drug combination in the parental models and a reduced combination effect in the resistant models (Fig. 4C–F). Little combination effect was seen in spheroid models, with both VIC-1911 and adavosertib eliminating most cell growth at the doses used (Fig. 4G and H). In contrast, in the longer-term clonogenic assays, the drug combination was much more effective than either single agent in parental cells and the AfaR and ErlR derivatives (Fig. 4I and J).

**Figure 5.**
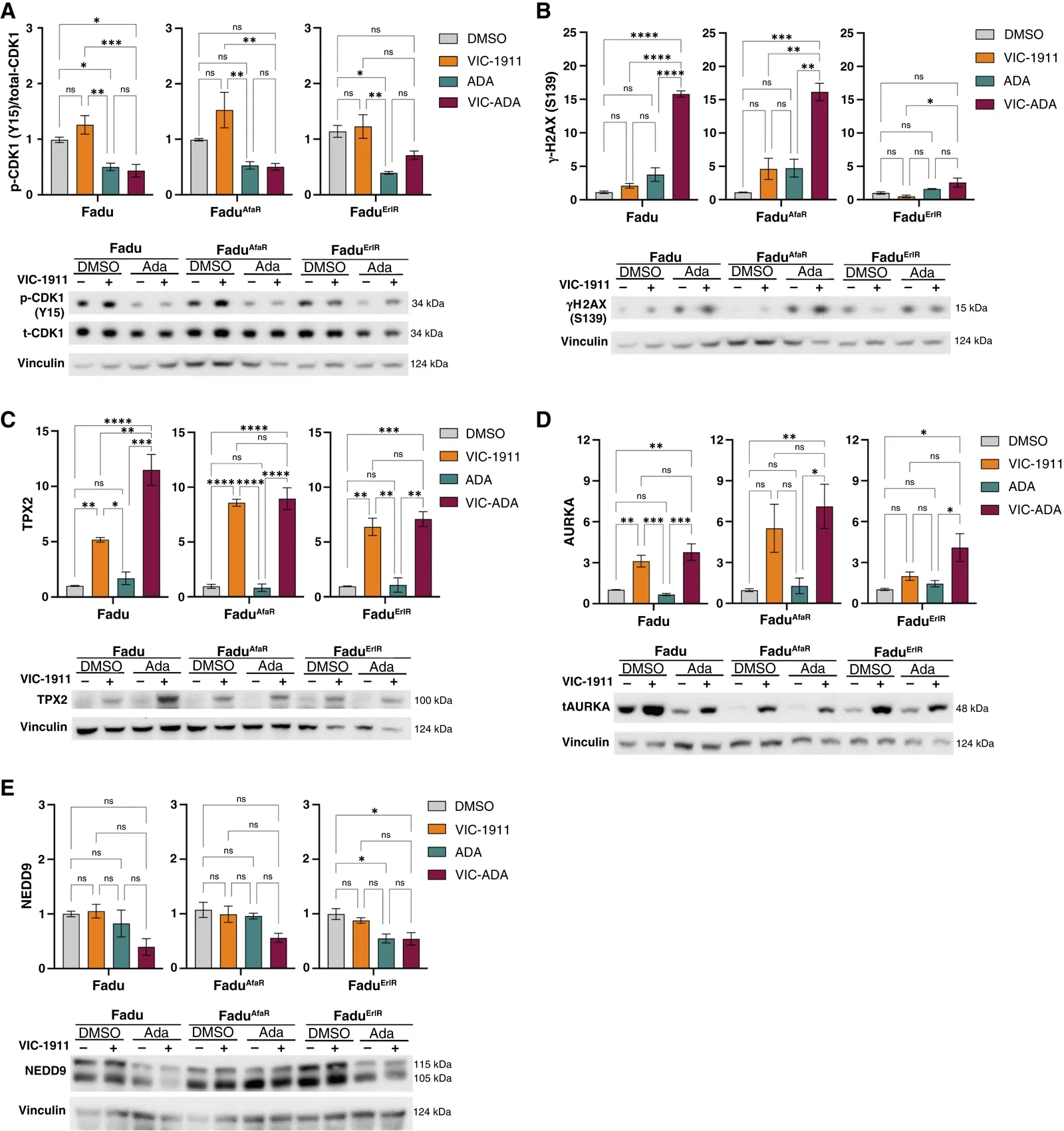
Signaling changes induced by treatment with VIC-1911 and adavosertib in parental, AfaR, and ErlR HNSCC models. **A–E,** Representative Western blots and quantification for phospho-CDK1 (Y15) and total-CDK1 (**A**), γH2AX (S139; **B**), TPX2 (**C**), total AURKA (**D**), and NEDD9 (**E**) in FaDu parental, ErlR, and AfaR cell models treated with DMSO, VIC-1911 (0.25 μmol/L), adavosertib (0.5 μmol/L), or combination for 24 hours. **A** and **B** share the same loading control, as they were derived from the same gel. Bar graphs display data normalized to the DMSO of each cell line. *P* values are based on one-way ANOVA followed by the Tukey multiple comparison test. *, *P* ≤ 0.05; **, *P* ≤ 0.01; ***, *P* ≤ 0.001; ****, *P* ≤ 0.0001. Data are shown as mean ± SEM of three biological replicates.

Signaling analysis at 24 hours after drug treatment demonstrated the effectiveness of adavosertib in decreasing levels of the inhibitory CDK1^Y15^ phosphorylation in all models, indicating the removal of G_2_–M checkpoints (Fig. 5A; Supplementary Fig. S6B). In contrast, adavosertib did not induce consistent changes in γH2AX staining (reflective of DNA damage) or expression of TPX2, NEDD9, or total AURKA across the parental or resistant models (Fig. 5B–E; Supplementary Fig. S6C–S6F). Single-agent VIC-1911 significantly induced CDK1^Y15^ phosphorylation in the Cal27 series of models, although not in the FaDu models, and had a limited effect on γH2AX or NEDD9 expression but induced TPX2 and total AURKA in most of the models (Fig. 5; Supplementary Fig. S6). In the FaDu series of models, the drug combination resulted in a significant elevation of γH2AX staining; this was also seen in Cal27^ErlR^, but not in parental Cal27 or Cal27^AfaR^ cells.

### *In vivo* analysis of AURKAi + WEE1i combination in parental versus AfaR tumors

**Figure 6.**
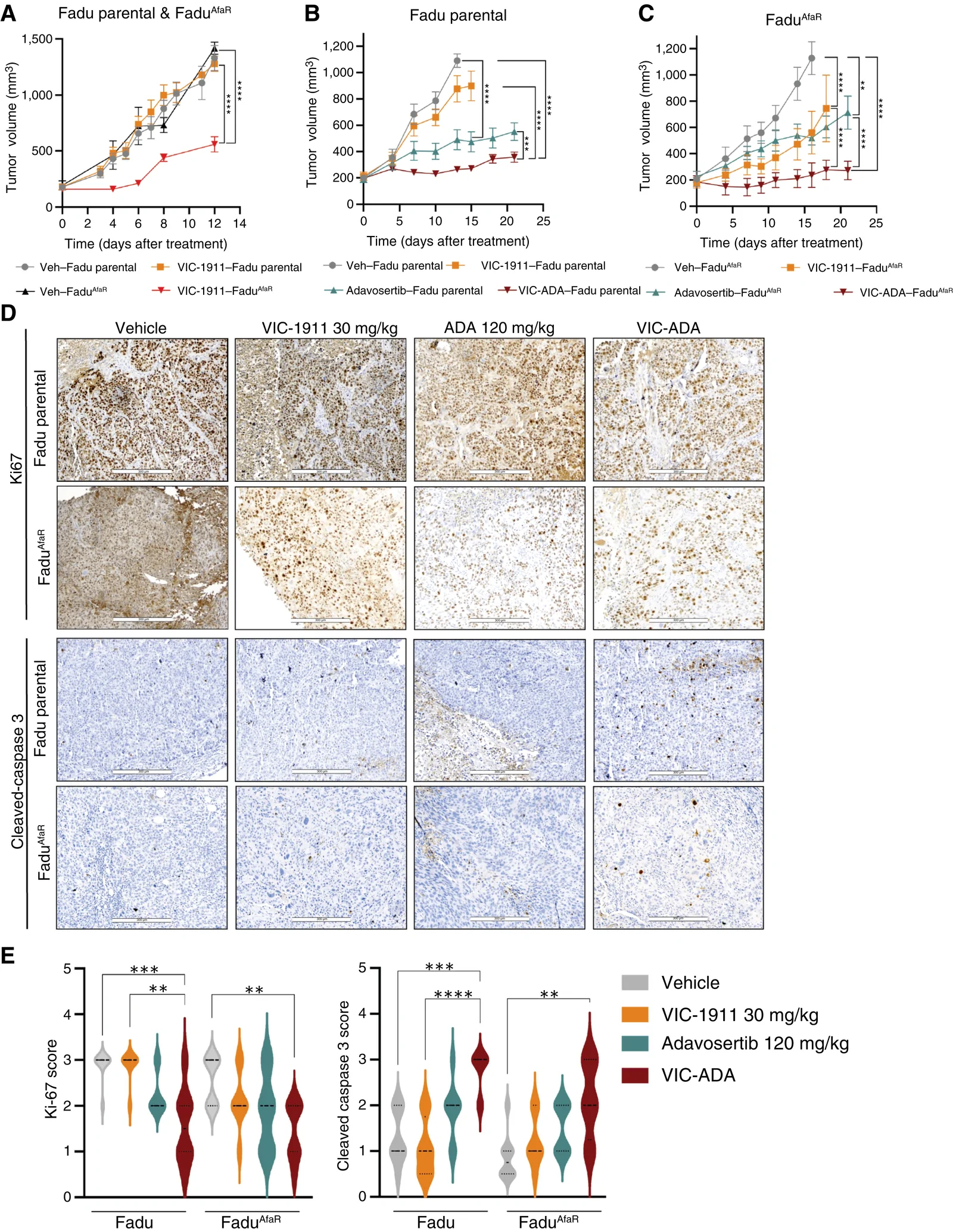
*In vivo* response of FaDu parental and Fadu^AfaR^ xenografts to VIC-1911 + adavosertib drug combination. **A,** Mice harboring either FaDu parental or FaDu^AfaR^ xenograft tumors were daily treated with vehicle (*n* = 8 for parental, *n* = 5 for AfaR) or VIC-1911 60 mg/kg (*n* = 8 for parental, *n* = 5 for AfaR) for 12 days to compare response to monotherapy. **B** and **C,** Mice harboring FaDu parental (**B**) or Fadu^AfaR^ (**C**) xenografts were daily treated with vehicle (*n* = 7), adavosertib 120 mg/kg (*n* = 7), VIC-1911 30 mg/kg (*n* = 8), or combination (*n* = 8) for 21 days. *P* values are based on two-way ANOVA followed by the Tukey multiple comparison test. **D** and **E,** Representative IHC staining images (**D**) and quantification (**E**) of Ki-67 and cleaved caspase 3 in FaDu parental or FaDu^AfaR^ xenograft tumors treated with the indicated drugs. Mice harboring FaDu parental or FaDu^AfaR^ xenografts were daily treated with vehicle (*n* = 7), adavosertib 120 mg/kg (*n* = 7), VIC-1911 30 mg/kg (*n* = 8), or combination (*n* = 8) for 21 days. Scale bars, 300 μm. Violin plots display median (-) and quartiles (…). *P* values are based on two-way ANOVA followed by the Tukey multiple comparison test. Only significant comparisons are displayed in the violin plots. *P* = 0.0332; **, *P* = 0.0021; ***, *P* = 0.0002; ****, *P* ≤ 0.0001.

To assess whether the VIC1911 + adavosertib combination was effective in the resistance setting, we used the FaDu parental and Fadu^AfaR^ models in xenograft analysis (Fig. 6). We first compared the response of these models to VIC-1911 at doses of 60 mg/kg (Fig. 6A), beginning dosing when xenografts were 100 to 150 mm^3^, and dosing for up to 12 days. Similar to results observed in clonogenic assays, the resistant Fadu^AfaR^ cells were significantly more sensitive to single-agent VIC-1911 at the higher dose compared with single-agent VIC-1911 at 30 mg/kg. We therefore assessed the combination effect using a lower VIC-1911 dose to increase the likelihood of enhancing a drug interaction.

Mice bearing either FaDu or FaDu^AfaR^ subcutaneous xenograft tumors were treated daily for up to 3 weeks with vehicle, adavosertib 120 mg/kg, VIC-1911 30 mg/kg, or the combination. Under these conditions, single-agent VIC-1911 did not significantly reduce growth in the FaDu parental model, but it did so in the FaDu^AfaR^ model (adjusted *P* < 0.0001). Adavosertib caused a significant reduction in growth in both FaDu parental (adjusted *P* < 0.0001) and resistant (adjusted *P* = 0.0021) cells (Fig. 6B and C). Importantly, the drug combination was extremely effective in both the EGFRi-sensitive and EGFRi-resistant settings, completely eliminating tumor growth over the time of the study in both models. Tumor control with the drug combination was significantly greater than treatment of cells with either single-agent adavosertib (adjusted *P* = 0.0002 for parental and *P* < 0.0001 for AfaR) or VIC-1911 (adjusted *P* < 0.0001 for both parental and AfaR).

IHC staining for recovered tumors indicated a significantly greater reduction in Ki-67 staining and elevation of staining for cleaved caspase 3 in FaDu^AfaR^ cells treated with 60 mg/kg VIC-1911, whereas no such effect was seen in FaDu parental cells (Supplementary Fig. S7A and S7B). In contrast, although neither 30 mg/kg VIC-1911 nor adavosertib significantly affected Ki-67 or cleaved caspase 3 when used as single agents, the combination significantly reduced Ki-67 and elevated caspase cleavage in both FaDu^AfaR^ and FaDu models (Fig. 6D and E). For both the parental and resistance models, VIC-1911 enhanced the expression of total AURKA and TPX2, whereas the use of the VIC-1911 + adavosertib combination strongly enhanced the expression of these two proteins (Supplementary Fig. S7C and S7D). Finally, single-agent use of drugs or drug combinations was well tolerated in all *in vivo* experiments, causing no loss of body weight or signs of distress throughout the study (Supplementary Fig. S8A–S8C).

## Discussion

This study is the first to explore the use of AURKAi VIC-1911 alone or in combination in the setting of EGFRi- or panERBBi-sensitive versus resistant HNSCC. The four new resistance models developed for this study have features of pEMT and/or upregulated TGFβ signaling, associated with clinical resistance to afatinib (43). A key finding of this study is the considerable difference in AURKA activity in the parental versus resistant models in short- versus longer-term growth models. Single-agent and combination activities of VIC-1911 in the reduction of viability and induction of apoptosis were reduced in resistant versus parental models, but single-agent activity in reducing viability was elevated in longer-term clonogenic analysis and *in vivo*. Importantly, the retained or heightened sensitivity to AURKA inhibition nominated the use of a VIC-1911 + adavosertib drug combination, which showed a useful combination effect both *in vitro* and *in vivo*, suggesting clinical utility in individuals with ERBBi resistance.

The data presented here highlight a number of differences from a prior report on the use of AURKA inhibition in EGFRi-resistant EGFR^mut^ NSCLC (21). For example, TPX2 is a well-established AURKA partner, stabilizer, and activator (38, 44). Although drug resistance associated with pEMT has been linked to elevated drug efflux and hence reduced intracellular drug dose levels (45), we observed comparable induction of TPX2 following AURKA treatment in parental and resistant cell models, implying that VIC-1911 is present at similar levels in both parental and resistant models. However, although transient inhibition of AURKA led to a compensatory induction of TPX2, we observed no basal upregulation of TPX2 or AURKA expression or AURKA activity in the resistant cell models.

In contrast, we did observe a common basal upregulation of NEDD9 in the resistant models. NEDD9, also known as HEF1 and CASL, is a scaffolding protein with complex activity in both the regulation of the cell cycle through association with AURKA and in the control of cancer migration and invasion (reviewed in refs. 46, 47). NEDD9 is both upregulated in and a known promoter of EMT (40, 41), suggesting it may be particularly relevant to the control of AURKA targeting to specific substrates or resistance to AURKAis in resistant cells. We note that our data do not indicate an elevated level of AURKA kinase activity in the resistant models. It is likely that the greater dependence on AURKA in these models represents a case of acquired addiction in the context of impaired EGFR/ERBB signaling (e.g., as in ref. 48), perhaps mediated by NEDD9-dependent interactions (39). Such acquired dependence has been frequently reported in studies of synthetic drug–drug and drug–gene interactions (49, 50).

We also note that we did not observe any effect of AURKA inhibition in the resistant models on the activation of the ERBB effectors AKT or ERK, or on BIM_EL_ expression (described as an important mediator of resistance in EGFR-mutated NSCLC; ref. 21). One possible explanation for the lack of BIM_EL_ engagement relates to intrinsic differences between EGFRi resistance in NSCLC versus HNSCC or in the EGFR^mut^ versus EGFR-overexpressing setting. These differences may be related to an altered signaling environment associated with pEMT and/or TGFβ; pEMT is associated with numerous signaling changes that promote resistance to ERBB family inhibition, affecting the NOTCH, HIPPO, and other pathways (36). The particularly strong baseline resistance observed in CAL27 cells may additionally reflect high levels of cMET expression or the presence of noncanonical activating mutations in *NRAS*.

Also, in contrast to NSCLC, in which EGFR pathway signaling is activated by mutation of the EGFR gene (51), in HNSCC the pathway is induced by EGFR overexpression (52). In cell lines derived from HNSCC tumors, erlotinib induces cell death with an IC_50_ based on short-term viability assays from 1.56 to 6.6 μmol/L (53); the IC_50_ of afatinib varies over a broad range in head and neck cell lines, ranging from 10 nmol/L to 8 μmol/L in short-term viability assays (54). Given these sometimes high baselines, selection for resistance typically reflects the requirement for much higher levels of drug to observe a growth inhibitory effect than is observed in lung models, with doses of erlotinib at 10 μmol/L or higher reported (55, 56). Depending on the cell line model, only a limited increase in IC_50_ may be observed between parental and resistance-selected models; further, at these high concentrations, the observed consequences of drug activity may not be solely confined to inhibition of the EGFR target.

The data emphasize the importance of using longer-term assays and comparing results across multiple assays for the study of AURKA activity. Viability assays typically showed less growth inhibition and cell death associated with VIC-1911 treatment than did longer-term assays, even when higher drug doses were used. This most likely reflects the fact that inhibition of AURKA causes cell-cycle arrest in G_2_- or M-phase; compensatory mechanisms, including checkpoint inhibitors, can sustain cell viability for some period of time but eventually lose efficacy, causing cells to enter mitosis with defects in the spindle apparatus, as reported in previous studies. Not all cells are initially affected by these inhibitors (i.e., some cells will have just completed mitosis and are not subject to drug effects until again entering M-phase after 24 hours). Cells can remain in mitosis due to triggered checkpoints for many hours. Furthermore, the disruption of mitosis by an AURKAi is stochastic; even after checkpoints fail, some cells will progress through mitosis with a tolerable amount of aneuploidy (57). Over time, as surviving cells with inhibited AURKA progress through two or three cycles, this leads to significant mitotic defects that trigger mitotic catastrophe in many cells (as in ref. 27). Viability assays, performed at 3 days, occur while checkpoints and nonlethal aneuploidy have allowed many cells to persist; in the longer-term clonogenic assays, checkpoint activity has been overcome, and cells begin to die. Notably, constitutive reduction in AURKA expression via genetic loss of one copy of AURKA leads, over time, to the formation of cancers associated with significant aneuploidy (57).

Differences are also observed between distinct longer-term assays, with greater efficacy seen in spheroid than in clonogenic assays. This may reflect a particular importance of AURKA in a 3D context, as has been suggested by some experiments (e.g., ref. 58). Alternatively, it may reflect greater sensitivity of CellTiter-Glo measurements versus staining of colonies in discriminating cell death. Similarly, the level of cell death observed *in vivo* in treated mice supports the idea of a potent, longer-term effect of the administration of the VIC-1911 combination. Among the limitations of this study, we were unable to evaluate whether the Cal27AfaR cell models behaved similarly to the Fadu^AfaR^ model in xenograft analysis, as the Cal27AfaR did not form solid tumors over an extended period.

Taken together, these results emphasize the fact that although much emphasis has been placed on the potential benefit of therapeutically disrupting the interaction between AURKA and TPX2 (59), AURKA interacts with a large number of proteins that positively and negatively affect its expression and activity (60). Moreover, some intriguing evidence suggests that distinct AURKAis differentially affect its interaction with various cellular partners (61), and some of these additional partners, such as MYC, may be valuable therapeutic targets in resistant HNSCC (62). HNSCC prognosis and survival are also strongly related to pathogenic mutations or epigenetic loss of tumor suppressors, including TP53 and CDKN2A (63). As p53 mutation causes increased expression of AURKA, contributing to mitotic abnormalities (64), cells with more highly compromised TP53 function may be particularly susceptible to treatments including AURKAis. We note that some AURKAis, such as alisertib and VIC-1911, also inhibit other Aurora family members, such as AURKB, with lesser potency; however, these drugs maintain a very strong bias toward AURKA > AURKB (IC_50_ 1.2 nmol/L versus 395 nmol/L for alisertib (65), and 1 nmol/L versus 95 nmol/L for VIC-1911 in enzymatic assays), making it very likely that activities reported here and previously (27) reflect targeting of AURKA. Recently reported drugs with even stronger selectivity for AURKA > AURKB (1,500-fold) maintain G_2_–M delay and cell death phenotypes (66), further supporting this interpretation.

More broadly, the use of multiple resistance cell models derived from parental Cal27 and FaDu cell lines highlighted the heterogeneous nature of drug resistance seen in the clinic, with evaluation of signaling consequences not being equivalent across the various models employed. Therefore, a striking result of this work is the maintained effectiveness of a WEE1 + AURKAi combination in a resistance setting. Although efficacy is attenuated compared with efficacy in the parental cell models, this has potentially valuable clinical implications, given the limited number of therapies showing any effectiveness in patients with resistance to EGFR or pan-HER inhibitors. Clinical use of AURKAis has yielded mixed results in the past, but with newer generation agents, it is yielding some promising results, including in the setting of cancers with implicated EGFR driver activity (e.g., ref. 67). Similarly, although the use of adavosertib has been somewhat limited by adverse events in clinical trials (68), exploration of dosing levels and schedules, paired with the definition of therapeutic biomarkers, and the development of next-generation WEE1is such as azenosertib (69) offer promise for the productive exploitation of WEE1 as a target. Given the lack of useful therapies for patients with acquired resistance to EGFRis, the results presented here suggest there may be value in an AURKAi plus WEE1i inhibitor combination in the clinical setting of recurrent/resistant HNSCC.

## Supplementary Material

Supplementary Table S1 Antibodies used in the study for western blotting

Supplementary Table S2 Differential expression analysis table deseq2

Supplementary Figure S1 Characterization of erlotinib-resistant (ErlR) and afatinib-resistant (AfaR) HNSCC cell models.

Supplementary Figure S2 Characterization of baselines and biological response to AURKA inhibition in parental, AfaR, and ErlR HNSCC cell lines.

Supplementary Figure S3 Cell cycle profiles of Fadu parental and AfaR cell models after 1, 3, or 5 days treatment with VIC-1911

Supplementary Figure S4 Signaling consequences in Fadu parental, AfaR, or ErlR cells of treatment with VIC-1911 alone or in combination with erlotinib or afatinib

Supplementary Figure S5 Signaling consequences in Cal27 parental, AfaR, or ErlR cells of treatment with VIC-1911 alone or in combination with erlotinib or afatinib

Supplementary Figure S6 Cell cycle and signaling changes induced by treatment with VIC-1911 and adavosertib in parental, AfaR, and ErlR HNSCC models.

Supplementary Figure S7 Combined AURKA and WEE inhibition reduces Ki-67, and elevates caspase-3, AURKA and TPX2 expression in xenograft tumors.

Supplementary Figure S8 Body weight of treated mice.

## Data Availability

The RNA-seq raw data and processed files are available in the NCBI GEO under the accession number GSE324492. All other data are available from the corresponding author upon request.
