# Supplementary material for: Combination AURKA and WEE1 Inhibition Exhibits Efficacy in EGFR or Pan-ERBB Inhibitor–Resistant Head and Neck Squamous Cell Carcinoma
Source: Cancer Res Commun. 2026 Aug 13;6(8):1916–32. doi: 10.1158/2767-9764.CRC-26-0368 (PMC13489673; doi:10.1158/2767-9764.CRC-26-0368)
Supplement: Supplementary file 1 [file crc-26-0368_supplementary_table_s1_suppst1.docx]

**Supplementary Table S1. Antibodies used in the study for western blotting.**

| **Antibody** | **Company** | **Catalog number** | **RRID** | **Dilution** |
| --- | --- | --- | --- | --- |
| pEGFR (T1068) | Cell Signaling Technology | 3777 | AB_2096270 | 1: 1,000 |
| total EGFR |  | 2646 | AB_2230881 | 1: 10,000 |
| pERK (T204) |  | 9101 | AB_331646 | 1: 1,000 |
| total ERK |  | 4695 | AB_390779 | 1: 10,000 |
| pAKT (S473) |  | 9271 | AB_329825 | 1: 1,000 |
| total AKT |  | 4691 | AB_915783 | 1: 1,000 |
| TPX2 |  | 8559 | AB_10827636 | 1: 1,000 |
| NEDD9 |  | 4044 | AB_1904002 | 1: 1,000 |
| pAURKA (Thr288), B (Thr232), C (Thr198) |  | 2914 | AB_2061631 | 1: 1,000 |
| total AURKA |  | 3092 | AB_2061342 | 1: 1,000 |
| pBIM (S69) |  | 4585 | AB_2065176 | 1: 1,000 |
| total BIM |  | 2933 | AB_1030947 | 1: 1,000 |
| pCDK1 (Tyr 15) |  | 9111 | AB_331460 | 1: 1,000 |
| p-γH2Ax (S139) |  | 9718 | AB_2118009 | 1: 1,000 |
| γH2Ax |  | 2595 | AB_10694556 | 1: 1,000 |
| PARP, cleaved PARP |  | 9542 | AB_2160739 | 1: 1,000 |
| Vimentin |  | 5741 | AB_10695459 | 1: 1,000 |
| pHER2 (Y1248) |  | 2247 | AB_331725 | 1: 1,000 |
| totalHER2 |  | 2165 | AB_10692490 | 1: 1,000 |
| E-cadherin |  | 3195 | AB_2291471 | 1: 1,000 |
| N-cadherin |  | 13116 | AB_2687616 | 1: 1,000 |
| Anti-Rabbit IgG |  | 2729 | AB_1031062 | 1: 500 |
| total CDK1 | GeneTex | GTX108120 | AB_1949938 | 1: 1,000 |
| Actin | Sigma-Aldrich | A5316 | AB_476743 | 1:10,000 |
| 2nd anti-rabbit HRP conjugated antibody | Cytiva | RA934V | AB_772206 | 1: 10,000 |
| 2nd anti-mouse IgG HRP conjugated antibody | Cytiva | NA931VS | AB_772210 | 1: 5,000 |
| 2nd anti-rabbit IgG AP conjugated antibody | Jackson Immunoresearch Labs | 711-055-152 | AB_2340591 | 1: 5,000 |
| 2nd anti-mouse AP conjugated antibody |  | 715-055-151 | AB_2340778 | 1: 5,000 |
